# Associations of human milk lactoferrin with measures of physical growth in very preterm infants

**DOI:** 10.1038/s41372-025-02447-2

**Published:** 2025-10-31

**Authors:** Paige K. Berger, Manasa Kuncham, Margaret L. Ong, Katherine Bell, Anne CC Lee, Sarbattama Sen, Annalee Furst, Lars Bode, Mandy B. Belfort

**Affiliations:** 1 Department of Pediatrics, Brigham and Women’s Hospital, Boston, MA USA; 2 Harvard Medical School, Boston, MA USA; 3 Department of Pediatrics, Warren Alpert School of Medicine, Brown University, Providence, RI USA; 4 Department of Pediatrics, Women and Infants Hospital of Rhode Island, Providence, RI USA; 5 Department of Pediatrics, Mother-Milk-Infant Center of Research Excellence (MOMI CORE), and Human Milk Institute (HMI), University of California, San Diego, La Jolla, CA USA

**Keywords:** Paediatrics, Outcomes research

## Abstract

**Objective:**

To determine associations of human milk lactoferrin concentrations with growth outcomes in very preterm infants.

**Study design:**

In 63 infants <32 weeks’ gestation, human milk samples on days 14 and 28 were analyzed for lactoferrin using electrochemiluminescence multiplex immunoassay, and values were averaged to generate mean concentrations. Anthropometry and body composition were measured at term-corrected age. Median regression was used to test median differences in outcomes across tertiles of mean human milk lactoferrin levels [lowest (0.06–0.13 mg/mL) vs. middle (0.14–0.22 mg/mL) and highest (>0.22 mg/mL)].

**Results:**

Compared to infants in the lowest tertile of human milk lactoferrin concentration, those in the highest tertile had greater head circumference z-score (β = 0.81 z-scores per mg/mL; 95% CI = 0.2, 1.4). There were no other differences between groups.

**Conclusion:**

Human milk lactoferrin concentrations were positively associated with head circumference z-scores during hospitalization. Future studies should examine the safety/utility of lactoferrin supplementation to optimize head growth.

## Introduction

Despite advancements in clinical care, very preterm infants (<32 weeks’ gestation) continue to fall behind the growth reference curves for healthy fetuses of the same gestational age [1]. These infants also have higher fat mass and lower fat free mass at term-corrected age than infants born at term, a pattern associated with adverse outcomes (e.g., neurodevelopmental deficits, cardiometabolic disease) [2, 3]. Importantly, the infant diet is a highly modifiable determinant of physical growth in the neonatal intensive care unit (NICU) and serves as a promising target to improve anthropometric and body composition outcomes [4]. A human milk diet is recommended for all very preterm infants due to its many medical benefits [5]. The protective value of human milk has been attributed to its myriad components, classified as macronutrients, micronutrients, and bioactives [6]. Prior studies have focused on human milk macronutrients, exposing gaps in protein and energy intakes that informed fortification practices for human milk in the NICU [7]. It is unclear whether other human milk components, specifically human milk bioactives, are also important for physical growth in very preterm infants.

Lactoferrin is the second most abundant protein in human milk and a bioactive with many functions [8]. Lactoferrin may affect physical growth through its role as a prebiotic, which supports the development of the gastrointestindal tract and maintenance of the gut microbiome [9, 10]. Animal studies, including models of intrauterine growth restriction, have shown that lactoferrin supplementation increased weight gain in rat pups and piglets, and found concomitant changes in the gut microbiome (e.g., increased growth of Bifidobacterium species) [11–13]. Moreover, an observational study in full-term and preterm infants found that greater concentrations of human milk lactoferrin were associated with greater amounts of Bifidobacterium species in stool samples [9]. Bifidobacterium species strengthen intestinal barrier function and break down macronutrients [14, 15]. These activities may ensure more efficient digestion/absorption to better support physical growth in very preterm infants.

Although there is compelling evidence in experimental models, human studies of lactoferrin and physical growth are limited to full-term infants and have reported inconsistent findings [16, 17]. One study in full-term infants found no association of human milk lactoferrin concentrations with anthropometric indices, while another found that greater intake was associated with greater fat mass and lower fat free mass (prior to adjustment for multiple comparisons) [16, 17]. Thus, the relationship between human milk lactoferrin concentration and physical growth outcomes in very preterm infants is still unknown. Therefore, the aim of this study was to determine associations of human milk lactoferrin concentration during NICU hospitalization with anthropometric and body composition outcomes in very preterm infants at term-corrected age. We hypothesized that greater human milk lactoferrin concentration would be associated with more favorable physical growth indices, including greater weight, length, head circumference, and fat free mass, indicative of overall larger body size and protein accretion [4].

## Materials and methods

### Participants

This ancillary study used data and biospecimens from a prospective observational study at Brigham and Women’s Hospital from 2015 to 2018, which assessed associations of human milk macronutrients with health outcomes in very preterm infants [18]. Inclusion criteria required infants to be born <32 weeks’ gestation and to be either singletons or twins. Eligible infants had to be ≤14 days at the time of enrollment and mothers had to plan on providing their own milk. Exclusion criteria were: 1) diagnosis of major congenital anomalies; 2) triplets or higher order multiples; and 3) unable to answer questions in English. The study protocol was approved by the Partners Human Research Committee and parents of participating infants provided written informed consent [18].

### Human milk collection and analysis

Almost all very preterm infants were predominantly fed refrigerated maternal milk, defined as ≥75% of enteral feeds derived from their mother’s own milk. As part of routine clinical care, mothers received standard written and verbal instructions to express milk using an electric breast pump and augment with hand expression at least 8 times/day. Bedside sampling of unfortified human milk for research was initiated once mothers produced enough to meet infant needs. One time/day, clinical nurses collected 3–5 mL maternal milk that was hand-warmed and gently mixed prior to collection. Samples may have been pooled for infant feeding from multiple maternal pumping sessions throughout the day, minimizing potential variation due to diurnal fluctuations. Samples may also have contained both foremilk and hindmilk. This approach was intended to yield samples most closely representing the infants’ actual diet in the NICU. Human milk samples were refrigerated at 4 °C for up to 3 days until analysis for macronutrient content in the parent study [18]. The remaining maternal milk that was not used in macronutrient analysis was frozen at −80 °C. From those samples, we identified human milk fed on 14 ± 7 days chronological age (timepoint 1) and 28 ± 7 days chronological age (timepoint 2), which typically reflect when very preterm infants transition to or have achieved full enteral feeds.

Lactoferrin concentrations (mg/mL) were quantified via electrochemiluminescence multiplex immunoassay technology using the mesoscale discovery platform lactoferrin U-plex assay [19, 20]. Both the capture antibody and the detection antibody are monoclonal mouse anti-human antibodies. The assay was validated to ensure that serial dilution of whole human milk samples produced parallelism in data, indicating that other components in human milk (e.g., lipids) do not interfere with the measurement. An advantage of using whole human milk is that is can be used without delipidating, a process that can introduce variability. It was expected that lactoferrin concentrations in whole human milk would be lower than those obtained from delipidated samples. Due to the high sensitivity of the assay, samples were diluted to 1:1,000,000 to remain within the dynamic range of the assay. To minimize errors associated with manual pipetting at such high dilutions, we used a robotic liquid handling system (Assist Plus/Voyager, Integra Biosciences, Hudson, NH) to maintain a coefficient of variation (CV) < 5%. Lactoferrin spike recovery was 98.4 ± 2.3%. All samples were analyzed at least in duplicate. The results are presented in mg/mL in whole human milk. The average lactoferrin concentration from two separate collection time points was calculated to provide a comprehensive measure of lactoferrin concentration for each infant [19].

### Anthropometry and body composition

We collected anthropometric measures as close as possible to term equivalent age from the medical record. Body weight was measured by clinical nurses to the nearest 1 g using calibrated digital scales (Scale-Tronix, Inc, White Plains, NY). Body length was measured by a trained registered dietitian to the nearest 0.1 cm with a recumbent length board using the 2-person method. Head circumference at the largest frontal occipital plane was measured by the same registered dietitian to the nearest 0.1 cm using non-stretchable tape. Anthropometric measures were used to calculate z-scores for postmenstrual age (PMA) and sex using the Fenton growth reference [21].

Body composition was assessed using air displacement plethysmography (PEAPOD, COSMED, Concord, CA, USA) for infants who could remain clinically stable off supplemental oxygen or other respiratory support for at least 5 min [2]. This device uses whole body densitometry to estimate fat mass, fat free mass, and body fat percent in infants weighing 1–8 kg and is approved for use in the NICU. The PEAPOD was zeroed with any equipment that could not be removed during the assessment, such as feeding tubes and oxygen cannulas. Body composition estimates were used to calculate z-scores for PMA using the Norris reference [2, 22].

### Collection of potential covariates

Maternal and very preterm infant characteristics at birth were abstracted from the electronic medical record using definitions provided by the Vermont Oxford Network [23]. Variables included sociodemographic and clinical data, such as infant sex, multiple gestation, and gestational age based on obstetrical estimate. Birth weight, length, and head circumference were recorded and used to calculate z-scores based on the Fenton growth reference [21]. Daily intake of maternal milk was also documented, including volume of intake, energy, and macronutrient content. Average maternal milk protein intake was calculated as the sum of daily measured maternal milk protein concentration multiplied by ingested volume, and divided by infant body weight, as previously described [7]. We also recorded the presence of co-morbidities, including necrotizing enterocolitis with Bell Stage 2 or higher, intraventricular hemorrhage Grade 3 or 4, and late-onset sepsis based on positive culture, which were based on standard definitions, and receipt of postnatal steroids [23].

### Statistical analysis

Descriptive statistics are presented as median (IQR) for continuous variables and frequency for categorical variables. Average human milk lactoferrin concentrations were used as the independent variable, categorized into ordinal measures to normalize skewed data. Specifically, human milk lactoferrin concentrations were grouped into the lowest (0.06–0.13 mg/mL), middle (0.14–0.22 mg/mL), and highest (>0.22 mg/mL) tertiles of concentration, with the lowest tertile serving as the reference group. Anthropometrics (weight, length, head circumference z-scores) and body composition indices (fat mass, fat free mass, body fat percent z-scores) at term-corrected age were used as dependent variables. Median differences in anthropometric and body composition indices between the lowest and the middle and highest tertiles were determined in separate models using median regression analysis.

Model 0 was unadjusted, Model 1 was adjusted for gestational age at birth, birthweight z-score, sex, and PMA at measurement. We adjusted for birth size, rather than calculating change in outcomes because this approach preserves statistical power and reduces potential bias from measurement error, which is particularly important given the challenges of accurately assessing birth size in very preterm infants due to clinical instability. Model 2 was adjusted for the same variables as Model 1 plus mean total protein intake (g/kg/day), which was correlated with mean human milk lactoferrin levels (*r* = 0.42, *p* = 0.001) [19]. We adjusted for birthweight z-score rather than birth length or head circumference z-scores because birthweight is a more standardized measure obtained with an electronic scale at the time of delivery. Covariates were selected based on prior knowledge of their associations with human milk composition and physical growth in very preterm infants [24, 25]. All models accounted for intrafamilial correlation among twins. SAS software was used for descriptive statistics. STATA was used for quantile regression (*P* < 0.05).

## Results

There were 103 very preterm infants whose parents provided written informed consent for their infant(s) to participate. Of those infants, we excluded 2 who died, 4 who were diagnosed with congenital anomalies after enrollment, and 4 who were transferred to a different hospital before outcomes were ascertained. Of the remaining 93 infants, 68 had maternal milk samples collected at 14 ± 7 days chronological age (timepoint 1) and 28 ± 7 days chronological age (timepoint 2) for lactoferrin analysis. Specifically, 62 had samples at both timepoints, 3 had a sample at timepoint 1 only, and 3 had a sample at timepoint 2 only. Of these 68 infants, 63 had available anthropometric outcomes and 41 had available body composition outcomes.

Participant characteristics are reported in Table 1 as median (IQR) or percent (%). Mothers were 33 (27, 36) years old at the time of delivery and had a pre-pregnancy BMI of 28.1 (23, 35) kg/m^2^. Seventy-nine percent self-identified as non-Hispanic and 42% self-identified as White. Very preterm infants were born at 28.5 (27, 30) weeks’ gestational age, had a birthweight of 1068 (858, 1458) grams, and consumed an average of 125 (120, 132) mL/kg/day. The average volume of human milk consumed did not significantly differ across tertiles of human milk lactoferrin concentration (*P* = 0.50). Anthropometric measures were collected at 38 (36, 40) weeks’ PMA and body composition measures were collected at 39 (37, 40) weeks’ PMA. There were no significant differences between the PMA at the anthropometrics assessment and the PMA at the body composition assessment (*P* = 0.88). The volume of human milk consumed did not significantly differ across tertiles of human milk lactoferrin concentration (*P* = 0.50).

**Table 1:** Participant characteristics.

Mothers (*N*)	Total	Tertile 1 0.06–0.13 mg/mL	Tertile 2 0.14–0.22 mg/mL	Tertile 3 >0.22 mg/mL	*P*^a^
	64	19	23	22	
Age at delivery, years	33.0 (27, 36)	34.0 (29, 39)	31.0 (27, 36)	28.5 (26, 35)	0.16
Lactoferrin, mg/mL^b^					
Time 1^c^	0.16 (0.1, 0.2)	0.11 (0.1, 0.1)	0.16 (0.2, 0.2)	0.24 (0.2, 0.3)	<0.01
Time 2^c^	0.18 (0.1, 0.3)	0.09 (0.1, 0.1)	0.17 (0.1, 0.2)	0.29 (0.3, 0.4)	<0.01
Mean^c^	0.18 (0.1, 0.2)	0.10 (0.1, 0.1)	0.18 (0.2, 0.2)	0.26 (0.2, 0.3)	<0.01
Race, *N* (%)^d^					<0.01
Asian	3 (5)	0 (0)	2 (9)	1 (5)	
Black	20 (31)	3 (16)	8 (35)	9 (41)	
White	27 (42)	15 (79)	6 (26)	6 (27)	
Other/unknown	14 (22)	1 (5)	7 (30)	6 (27)	
Hispanic, *N* (%)^d^	13 (21)	0 (0)	5 (24)	8 (36)	0.01
Infants (*N*)	68	22	23	23	
Birthweight, g	1068 (858, 1458)	955 (650, 1470)	1060 (865, 1420)	1260 (970, 1460)	0.33
Birthweight, z-score	−0.09 (−0.6, 0.4)	−0.01 (−0.9, 0.4)	−0.25 (−1.1, 1.9)	0.09 (−0.5, 0.4)	0.56
Birth length, cm	36.6 (34, 40)	35.7 (31, 40)	36.4 (34, 40)	37.0 (36, 40)	0.18
Birth length, z-score	−0.07 (−0.5, 0.6)	−0.11 (−0.6, 0.8)	−0.17 (−0.9, 0.6)	0.14 (−0.5, 0.6)	0.46
Birth HC, cm	25.5 (23, 28)	24.9 (22, 28)	25.5 (24, 28)	26.0 (25, 28)	0.32
Birth HC, z-score	−0.34 (−1.0, 0.2)	−0.31 (−1.1, 0.1)	−0.46 (−0.9, 0.1)	−0.29 (−1.1, 0.4)	0.78
GA, weeks	28.5 (27, 30)	27.8 (25, 30)	29.1 (27, 30)	28.6 (27, 31)	0.46
SGA, %^d^	10 (15)	5 (23)	4 (17)	1 (4)	0.19
Average human milk volume, mL/kg/day	125 (120, 132)	126 (121, 132)	122 (117, 128)	125 (120, 131)	0.50
Average human milk energy, kcal/kg/day	83.4 (78, 91)	84.0 (79, 92)	81.2 (74, 83)	88.5 (80, 92)	0.46
Average human milk protein, g/kg/day	1.55 (1.4, 1.9)	1.37 (1.2, 1.7)	1.57 (1.4, 1.8)	1.58 (1.6, 2.2)	0.04
Anthropometry PMA, weeks^e^	38.3 (36, 40)	39.7 (38, 41)	38.9 (37, 40)	38.0 (36, 40)	0.37
Body composition PMA, weeks^e^	39.4 (37, 40)	39.7 (37, 41)	39.4 (38, 40)	39.1 (38, 40)	0.70
Female, *N* (%)^d^	31 (46)	13 (59)	9 (39)	9 (39)	0.30
Twins, *N* (%)^2^	12 (18)	7 (32)	2 (9)	3 (13)	0.10
Comorbidities, *N* (%)^d^					
NEC	1 (2)	1 (6)	0 (0)	0 (0)	0.35
Severe IVH or PVL	5 (8)	2 (10)	2 (9)	1 (4)	0.12
Sepsis/meningitis	3 (5)	1 (5)	2 (9)	0 (0)	0.11
Steroids for CLD	10 (15)	5 (23)	3 (14)	2 (9)	0.35
Respiratory support	23 (43)	10 (53)	7 (44)	6 (33)	0.57

All values are median (IQR) or *N* (%).

Tertile 1 serves as the reference group.

*CLD* chronic lung disease, *GA* gestational age, *HC* head circumference, *IVH* intraventricular hemorrhage, *NEC* necrotizing enterocolitis, *PMA* postmenstrual age, *PVL* periventricular leukomalacia, *SGA* small for gestational age.

^a^ANOVA used to test for significant differences in continuous variables across tertiles of human milk lactoferrin.

^b^No significant differences in human milk lactoferrin concentrations between timepoints 1 and 2 (*P* = 0.70).

^c^Kruskal–Wallis used to test for significant differences across tertile groups and Wilcoxon rank sum test used to determine significant differences between tertile groups.

^d^Chi-square tests or Fisher’s exact test used to test for significant differences in categorical variables across tertiles of human milk lactoferrin.

^e^No significant differences in PMA at anthropometric assessment and body composition assessment (*P* = 0.88).

Concentrations of lactoferrin in whole human milk ranged from 0.06–0.49 mg/mL. Median differences in anthropometric outcomes between tertiles of human milk lactoferrin are presented in Table 2. In both the unadjusted and adjusted models, very preterm infants in the highest tertile of human milk lactoferrin concentration had greater head circumference z-scores compared to those in the lowest tertile. Specifically, after adjustment for gestational age, birthweight z-score, sex, PMA at measurement, and mean protein intake, participants in the highest tertile of human milk lactoferrin concentration had 0.81 (95% CI = 0.2, 1.4) greater head circumference z-score compared to those in the lowest tertile. Sensitivity analyses adjusting for birth head circumference z-score instead of birthweight z-score yielded consistent results (Model 1: β = 0.80, 95% CI = 0.2, 1.4; Model 2: β = 0.70, 95% CI = 0.0, 1.4) (Supplementary Tables 1 and 2). Similarly, excluding very preterm infants who developed necrotizing enterocolitis, severe intraventricular hemorrhage, and sepsis yielded comparable findings to those observed in the total sample. Weight length, and body composition z-scores did not differ by tertile of human milk lactoferrin concentration (Table 3).

**Table 2:** Differences in anthropometric measures between tertiles of human milk lactoferrin concentrations.^a,b,c^.

	Anthropometric Measures (Fenton Z-Scores; *N* = 63)^d^					
	Weight		Length		Head circumference	
	Tertile 2	Tertile 3	Tertile 2	Tertile 3	Tertile 2	Tertile 3
Model 0	β = 0.19 (−0.9, 1.3) *P *= 0.73	β = 0.74 (−0.2, 1.7) *P *= 0.14	β = −0.27 (−1.3, 0.7) *P *= 0.59	β = 0.14 (−0.8, 1.0) *P *= 0.76	β = 0.23 (−0.6, 1.1) *P *= 0.58	β = 0.87 (0.1, 1.6) *P *= 0.03
Model 1	β = 0.00 (−0.5, 0.5) *P *= 0.99	β = 0.38 (−0.1, 0.9) *P *= 0.13	β = 0.12 (−0.5, 0.8) *P *= 0.71	β = 0.55 (−0.1, 1.2) *P *= 0.07	β = 0.42 (−0.2, 1.1) *P *= 0.20	β = 0.79 (0.2, 1.3) *P *= 0.01
Model 2	β = 0.09 (−0.4, 0.6) *P *= 0.73	β = 0.41 (0.0, 0.8) *P *= 0.06	β = −0.05 (−0.9, 0.8) *P *= 0.91	β = 0.38 (−0.4, 1.1) *P *= 0.31	β = 0.43 (−0.2, 1.0) *P *= 0.16	β = 0.81 (0.2, 1.4) *P *= 0.01

^a^Median regression analysis accounting for intrafamilial correlation between twins. Values are reported as β (95% CI) and *P*-value.

^b^Model 0 is unadjusted; Model 1 adjusted for gestational age at birth, birthweight z-score, sex, and PMA; Model 2 adjusted for same variables as Model 1 plus mean protein intake.

^c^β estimates indicate median differences in body size z-scores compared with reference group of infants in the lowest tertile of human milk lactoferrin concentration (Tertile 1).

^d^For participants who did not have head circumference z-score collected at term-corrected age, measurements taken at NICU discharge were used in analyses.

**Table 3:** Differences in body composition measures between tertiles of human milk lactoferrin concentrations.^a,b^.

	Body composition (Norris Z-Scores (*N* = 41)^c^					
	Fat mass		Fat free mass		Body fat percent	
	Tertile 2	Tertile 3	Tertile 2	Tertile 3	Tertile 2	Tertile 3
Model 0	β = 0.68 (−0.6, 1.9) *P *= 0.28	β = 0.93 (−0.4, 2.3) *P *= 0.16	β = 1.02 (−1.1, 3.1) *P *= 0.34	β = 0.73 (−1.2, 2.6) *P *= 0.44	β = 0.10 (−1.0, 1.2) *P *= 0.85	β = 0.69 (−0.4, 1.8) *P *= 0.22
Model 1	β = 0.27 (−1.0, 1.5) *P *= 0.67	β = 0.62 (−0.6, 1.9) *P *= 0.32	β = 0.55 (−0.3, 1.4) *P *= 0.19	β = 0.40 (−0.4, 1.2) *P *= 0.30	β = −0.12 (−1.1, 0.9) *P *= 0.81	β = 0.71 (−0.4, 1.8) *P *= 0.21
Model 2	β = 0.28 (−1.0, 1.6) *P *= 0.67	β = 0.62 (−0.8, 2.1) *P *= 0.38	β = 0.54 (−0.2, 1.3) *P *= 0.14	β = 0.33 (−0.4, 1.0) *P *= 0.33	β = −0.11 (−1.2, 1.0) *P *= 0.84	β = 0.73 (−0.6, 2.1) 0.29

^a^Median regression analysis accounting for intrafamilial correlation between twins. Values are reported as β (95% CI) and *P*-value.

^b^Model 0 is unadjusted; Model 1 adjusted for gestational age at birth, birthweight z-score, sex, and PMA; Model 2 adjusted for same variables as Model 1 plus mean protein intake.

^c^β estimates indicate median differences in body composition z-scores compared with reference group in the lowest tertile of human milk lactoferrin concentration (Tertile 1).

## Discussion

In a cohort of very preterm infants, we found that greater human milk lactoferrin concentrations were associated with greater head circumference z-score, an anthropometric indicator of larger brain size that may predict better neurodevelopmental outcomes [4, 26]. There were no other associations of human milk lactoferrin concentrations with anthropometric or body composition outcomes at term-corrected age. Our observations are consistent with those from our prior study in the same cohort, which found that higher human milk lactoferrin concentrations were associated with larger total/regional brain volumes using magnetic resonance imaging (MRI) [19]. Collectively, our findings indicate that human milk lactoferrin may have a distinct role in promoting brain growth, rather than generally promoting overall larger body size and protein accretion, which indirectly impact the brain [2, 27].

Prior observational studies examining the influence of human milk lactoferrin concentration on physical growth outcomes focus exclusively on full-term infants [16, 17]. In full-term infants, human milk lactoferrin concentration at 6 months was not associated with weight, length, or head circumference z-scores at 12 months, while the concentration from 2 to 12 months was not associated with fat mass, fat free mass, or percent fat mass at 12 months (after adjusting for multiple comparisons) [16, 17]. Similarly, in our study of very preterm infants, there were no associations of human milk lactoferrin concentration with weight or length z-scores or body composition outcomes. However, we found a positive association of human milk lactoferrin with head circumference z-score. Moreover, the relationship between human milk lactoferrin and head circumference z-score persisted even after adjusting for birth head circumference z-score, indicating that human milk lactoferrin may contribute to postnatal head growth independent of initial head size. Differences in findings may be due to the unique vulnerability of very preterm infants compared to full-term infants [28]. Very preterm infants are at elevated risk for neuroinflammation, which may impair brain growth [29, 30]. Thus, these infants may be more sensitive or responsive to the neuroprotective properties of lactoferrin than their full-term counterparts.

Additionally, randomized controlled trials designed to test the effects of lactoferrin supplementation on infection-related indices in very preterm infants (which report mixed findings) have added anthropometrics as secondary outcomes; that approach was based in part on the hypothesis that the benefits of lactoferrin intake on complications such as necrotizing enterocolitis and sepsis may lead to improvements in physical growth [31–33]. Overall, those studies also reported null findings. In infants born at 28 weeks’ gestation, supplementation with recombinant human milk lactoferrin from 1 to 28 days had no effect on weight gain at NICU discharge [32]. In infants born at 30 weeks’ gestation, supplementation with bovine lactoferrin for 8 weeks had no effect on weight, length, or head circumference z-scores at 24 months [33]. While we also found no association of human milk lactoferrin concentration with weight or length z-scores, differences in findings related to head circumference z-score may be due to the timing of follow-up. Our study captured the impact of lactoferrin during the most dynamic period of brain growth, coinciding with NICU hospitalization, and may better reflect its effects before exposure to confounding factors in the post-discharge environment at 24 months [34, 35].

Although head circumference z-score is a non-specific indicator of brain size, our data supporting a positive association of human milk lactoferrin with head circumference z-score complement findings from our prior study using MRI to measure brain volume directly in the same cohort [19]. In that study, human milk lactoferrin concentration was associated with larger total brain, cortical gray matter, and deep gray matter volumes at term-corrected age [19]. Head circumference z-score in the present study also correlated with total brain volume in the prior study (r = 0.45, *p *= 0.01). Our findings lend additional support for the use of head circumference z-score as a valid and less resource-intensive proxy for brain size in the NICU [36].

It is plausible that the influence of lactoferrin on the brain is due to its function as a protein nutrient, which contributes essential amino acids to support structural development [37, 38]. Lactoferrin is an abundant protein in human milk, and higher protein intake is associated with greater head circumference and larger total/regional brain volumes in very preterm infants [18, 39, 40]. Yet, we found that the positive association of human milk lactoferrin concentration with head circumference z-score was independent of total protein intake, suggesting that lactoferrin may affect brain growth through processes beyond its role as a protein nutrient [41]. In addition, there was no association of human milk lactoferrin concentration with weight, length, or fat free mass z-scores, which reflect overall body size and protein accretion and are predictors of brain growth at NICU discharge [2]. Collectively, our data suggest that human milk lactoferrin may have functions that extend beyond simply providing essential amino acids [42, 43].

Indeed, we speculate that human milk lactoferrin may affect brain growth through its diverse bioactive functions, which may include prebiotic and anti-inflammatory properties that exert neuroprotective effects [41]. For instance, its prebiotic properties promote the growth of beneficial bacteria in the gastrointestinal tract, with far-reaching effects on the brain [10, 44]. In vitro, human milk lactoferrin stimulated the growth of Bifidobacterium species in cell culture [45, 46]. In very preterm infants, greater human milk lactoferrin concentration was associated with greater amounts of Bifidobacterium species in stool samples, and greater amounts of Bifidobacterium species were associated with larger head circumference [9, 47, 48]. The influence of Bifidobacterium species on head circumference may be due to its production of short-chain fatty acids (SCFA), which are byproducts of bacterial fermentation [49]. SCFA are absorbed by colonocytes and enter vagal pathways to the brain [50, 51]. SCFA are utilized as energy substrates to support brain metabolism and also regulate the expression of brain-derived neurotrophic factor (BDNF), a protein that supports the growth and proliferation of neurons and the strengthening of synaptic connections [50, 51].

Additionally, human milk lactoferrin may be protective against neuroinflammation that would otherwise impair brain growth [42]. Animal models of both hypoxic-ischemic brain injury and intrauterine growth restriction have shown that supplementation with bovine lactoferrin in rat pups decreased damage to cortical structures in the brain by reducing the expression of pro-inflammatory proteins (e.g., tumor necrosis factor-alpha, interleukin-6, Bcl-2) [52]. Supplementation with bovine lactoferrin in rat pups also decreased the expression of genes associated with glutamatergic excitotoxicity, a process that leads to neuronal damage and cell death [43]. The findings suggest that human milk lactoferrin may be neuroprotective by reducing pro-inflammatory proteins and may also preserve neuronal integrity, thereby supporting brain growth in vulnerable populations [43, 52].

Strengths of the study include its focus on very preterm infants, who are at elevated risk for suboptimal growth and stand to benefit most from human milk lactoferrin intake. We also used standard anthropometric measures along with detailed body composition assessments to provide a more comprehensive evaluation of physical growth. This study also had several limitations. The observational design precludes us from establishing causality. We measured human milk lactoferrin at only two timepoints, which may not fully account for temporal changes in concentration. To address this, we calculated mean human milk lactoferrin levels and divided values into tertiles to define thresholds that may help inform dosing strategies for future clinical trials. Larger studies with higher rates of co-morbidities are also needed to understand their impact on associations of human milk lactoferrin with physical growth.

Another potential limitation is that we measured human milk lactoferrin concentrations in whole human milk samples using electrochemiluminescence multiplex immunoassay, and our values were therefore lower than those reported in studies using delipidated human milk samples. However, there is no standardized analytic technique across studies, and even those that used similar methods to each other (e.g., enzyme-linked immunosorbent assay) have reported variable results, ranging from 0.4 mg/mL to 8.5 mg/mL [17, 53, 54]. Importantly, we found that human milk lactoferrin concentrations in this cohort of very preterm infants [median (IQR) = 0.2 (0.1–0.2) mg/mL] were very similar to those in a cohort of infants in rural Bangladesh [0.1 (0.1–0.2) mg/mL], which were both measured via electrochemiluminescence multiplex immunoassay in whole human milk samples [55].

In conclusion, greater human milk lactoferrin concentration was associated with greater head circumference z-score, but not other anthropometric or body composition indicators of physical growth. Our data highlight lactoferrin as a candidate to promote brain growth and development in very preterm infants and lend support to future clinical trials to test its neuroprotective effects and potential for supplementation in the NICU.

## Supplementary information

Supplementary Table 1 and 2. Differences in Anthropometric Measures Between Tertiles of Human Milk Lactoferrin Concentrations, Adjusted for Birth Length or Head Circumference Z-Scores

## Data Availability

The data that support the findings of this study are not openly available due to reasons of sensitivity and are available from the corresponding author upon reasonable request. Data are stored in controlled access data storage at Brigham and Women’s Hospital.
