# Supplementary material for: Associations of human milk lactoferrin with measures of physical growth in very preterm infants
Source: J Perinatol. 2025 Oct 31;46(3):403–9. doi: 10.1038/s41372-025-02447-2 (PMC13008772; doi:10.1038/s41372-025-02447-2)
Supplement: Supplementary file 1 — Supplementary Table 1 and 2. Differences in Anthropometric Measures Between Tertiles of Human Milk Lactoferrin Concentrations, Adjusted for Birth Length or Head Circumference Z-Scores [file 41372_2025_2447_MOESM1_ESM.docx]

**Supplementary Table 1. Differences in Anthropometric Measures Between Tertiles of Human Milk Lactoferrin Concentrations, Adjusted for Birth Length Z-Score.^a,b,c^**

|  | **Anthropometric Measures (Fenton Z-Scores; N=63)^d^** | | | | | |
| --- | --- | --- | --- | --- | --- | --- |
|  | **Weight** | | **Length** | | **Head Circumference** | |
|  | **Tertile 2** | **Tertile 3** | **Tertile 2** | **Tertile 3** | **Tertile 2** | **Tertile 3** |
| **Model 0** | β= 0.19  (-0.9, 1.3)  P= 0.73 | β= 0.74  (-0.2, 1.7)  P= 0.14 | β= -0.27  (-1.3, 0.7)  P= 0.59 | β= 0.14  (-0.8, 1.0)  P= 0.76 | β= 0.23  (-0.6, 1.1)  P= 0.58 | β= 0.87  (0.1, 1.6)  P= 0.03 |
| **Model 1** | β= 0.38  (-0.4, 1.2)  P= 0.34 | β= 0.68  (0.1, 1.3)  P= 0.03 | β= 0.09  (-0.7, 0.9)  P= 0.81 | β= 0.42  (-0.1, 1.0)  P= 0.14 | β= 0.44  (-0.3, 1.1)  P= 0.21 | β= 0.68  (0.1, 1.3)  P= 0.03 |
| **Model 2** | β= 0.10  (-0.5, 0.7)  P= 0.75 | β= 0.42  (-0.1, 1.0)  P= 0.14 | β= 0.15  (-0.6, 0.9)  P= 0.69 | β= 0.60  (-0.1, 1.3)  P= 0.08 | β= 0.47  (-0.3, 1.2)  P= 0.21 | β= 0.52  (-1.0, 1.2)  P= 0.10 |

^a^Median regression analysis accounting for intrafamilial correlation between twins. Values are reported as β (95% CI) and P-value.

^b^Model 0 is unadjusted; Model 1 adjusted for gestational age at birth, birth length z-score, sex, and PMA; Model 2 adjusted for same variables as Model 1 plus mean protein intake.

^c^β estimates indicate median differences in body size z-scores compared with reference group of infants in the lowest tertile of human milk lactoferrin concentration (Tertile 1).

^d^For participants who did not have head circumference z-score collected at term-corrected age, measurements taken at NICU discharge were used in analyses.

**Supplementary Table 2. Differences in Anthropometric Measures Between Tertiles of Human Milk Lactoferrin Concentrations, Adjusted for Birth Head Circumference Z-Score.^a,b,c^**

|  | **Anthropometric Measures (Fenton Z-Scores; N=63)^d^** | | | | | |
| --- | --- | --- | --- | --- | --- | --- |
|  | **Weight** | | **Length** | | **Head Circumference** | |
|  | **Tertile 2** | **Tertile 3** | **Tertile 2** | **Tertile 3** | **Tertile 2** | **Tertile 3** |
| **Model 0** | β= 0.19  (-0.9, 1.3)  P= 0.73 | β= 0.74  (-0.2, 1.7)  P= 0.14 | β= -0.27  (-1.3, 0.7)  P= 0.59 | β= 0.14  (-0.8, 1.0)  P= 0.76 | β= 0.23  (-0.6, 1.1)  P= 0.58 | β= 0.87  (0.1, 1.6)  P= 0.03 |
| **Model 1** | β= 0.17  (-0.6, 0.9)  P= 0.64 | β= 0.62  (0.0, 1.3)  P= 0.06 | β= -0.29  (-0.9, 0.3)  P= 0.35 | β= 0.15  (-0.5, 0.8)  P= 0.64 | β= 0.52  (-0.1, 1.1)  P= 0.83 | β= 0.80  (0.2, 1.4)  P= 0.01 |
| **Model 2** | β= 0.19  (-0.8, 1.1)  P= 0.71 | β= 0.37  (-0.4, 1.1)  P= 0.34 | β= -0.20  (-1.2, 0.8)  P= 0.69 | β= 0.13  (-0.7, 1.0)  P= 0.75 | β= 0.51  (-0.3, 1.3)  P= 0.20 | β= 0.70  (0.0, 1.4)  P= 0.06 |

^a^Median regression analysis accounting for intrafamilial correlation between twins. Values are reported as β (95% CI) and P-value.

^b^Model 0 is unadjusted; Model 1 adjusted for gestational age at birth, birth head circumference z-score, sex, and PMA; Model 2 adjusted for same variables as Model 1 plus mean protein intake.

^c^β estimates indicate median differences in body size z-scores compared with reference group of infants in the lowest tertile of human milk lactoferrin concentration (Tertile 1).

^d^For participants who did not have head circumference z-score collected at term-corrected age, measurements taken at NICU discharge were used in analyses.
